# Hypercalcemia Due to Paraneoplastic Production of 1,25- Dihydroxyvitamin D in a Young Female with Dysgerminoma

**DOI:** 10.7759/cureus.6097

**Published:** 2019-11-08

**Authors:** Iqra Iqbal, Muhammad Atique Alam Khan, Yasir Khan, Nishanth Thalambedu, Samavia Munir

**Affiliations:** 1 Internal Medicine, Abington Hospital - Jefferson Health, Abington, USA

**Keywords:** dysgerminoma, vitamin d, hypercalcemia

## Abstract

Humoral hypercalcemia of malignancy (HHM) can be caused by ectopic paraneoplastic production of 1, 25 dihydroxy vitamin D due to the hyperactivity of the 1 alpha-hydroxylase enzyme. We present a case of a 19-year-old female who was admitted with bilateral dysgerminomas and significant hypercalcemia. Hypercalcemia was initially managed medically and then resolved with the surgical resection of the tumors. Although most cases are attributed to a high parathyroid hormone-related peptide (PTHrP) and bone metastases, <1% of cases can result from paraneoplastic production of 1,25 dihydroxyvitamin D due to increased activity of 1 alpha-hydroxylase.This is one of the rare cases of hypercalcemia, which not only adds to the limited number of cases of hypercalcemia associated with dysgerminoma but also is the first case report showing that vitamin D can be a paraneoplastic factor itself.

## Introduction

There are a limited number of cases of dysgerminoma associated with hypercalcemia. In these cases, hypercalcemia was either associated with the production of the parathyroid hormone-related peptide (PTHrP) or other humoral factors. However, there is no prior documented case of hypercalcemia secondary to vitamin D overproduction [1]. We present a rare case of hypercalcemia due to ectopic paraneoplastic production of 1 alpha-hydroxylase and 1,25 dihydroxyvitamin D. Our case adds to the limited literature available on 1,25 dihydroxyvitamin D and 1 alpha-hydroxylase overproduction from dysgerminomas. We, therefore, advocate that this rare but possible cause of hypercalcemia should be kept in mind when investigating a case of dysgerminoma.

## Case presentation

A 19-year-old female presented to the hospital with complaints of abdominal distension and loss of appetite. She also experienced significant unintentional weight loss, which she was unable to quantify. Apart from this, she reported dyspnea on exertion, which she attributed to the abdominal distention. She denied chest pain and bowel or urinary symptoms and had no significant past medical and family history of any parathyroid hyperplasias. She was not taking any supplemental vitamin D.

On physical examination, her vitals were temperature 98.8 F, blood pressure 141/72, heart rate 121, respiratory rate 20, oxygen saturation 97% on room air. On clinical examination, the abdomen was distended, non-tender with palpable left lower quadrant abdominal masses. Bowel sounds were hypoactive, and there was no rebound tenderness and guarding. The neurological, cardiovascular, and pulmonary examination was unremarkable.

Lab investigations were remarkable for hypercalcemia, raised lactate dehydrogenase (LDH), deranged renal functions, and metabolic acidosis with respiratory compensation. The detailed laboratory investigations are shown in Table 1. Normal ranges are mentioned in parenthesis.

**Table 1:** Laboratory investigations for workup of hypercalcemia

Lab test name	Lab value	Lab test name	Lab value
Calcium:	16.1 (8.4- 10.2 mg/dl)	Lactate dehydrogenase (LDH)	7000 (100- 220 U/L)
Albumin	4 (3.3- 4.7 g/dL )	Cancer antigen 125 (CA 125)	2502 (0 - 37 U/mL)
25, hydroxyvitamin D level	30 (20-100 ng/ml)	Thyroid-stimulating hormone (TSH)	0.776 (0.35- 5.5 microIU/ml)
Creatinine	2.6 (0.6-1.2 mg/dl)	1,25, di-hydroxyvitamin D3	58 (18-72 pg/ml)
Carcinoembryonic antigen (CEA)	<1 (0.0- 5 ng/ml)	Cancer antigen 19-9 (CA 19-9)	5.18 (0- 37 U/ml)
Intact parathyroid hormone (PTH)	5.9 (25- 88 pg/ml)	Parathyroid hormone-related peptide (PTHrP)	14 (14-27 pg/ml)
Beta human chorionic gonadotrophin (B-HCG) with titer	<5 ( <5 MIU/ml)	Cortisol	14.7 (3.7- 19.4 microgram/dl)

**Figure 1:**
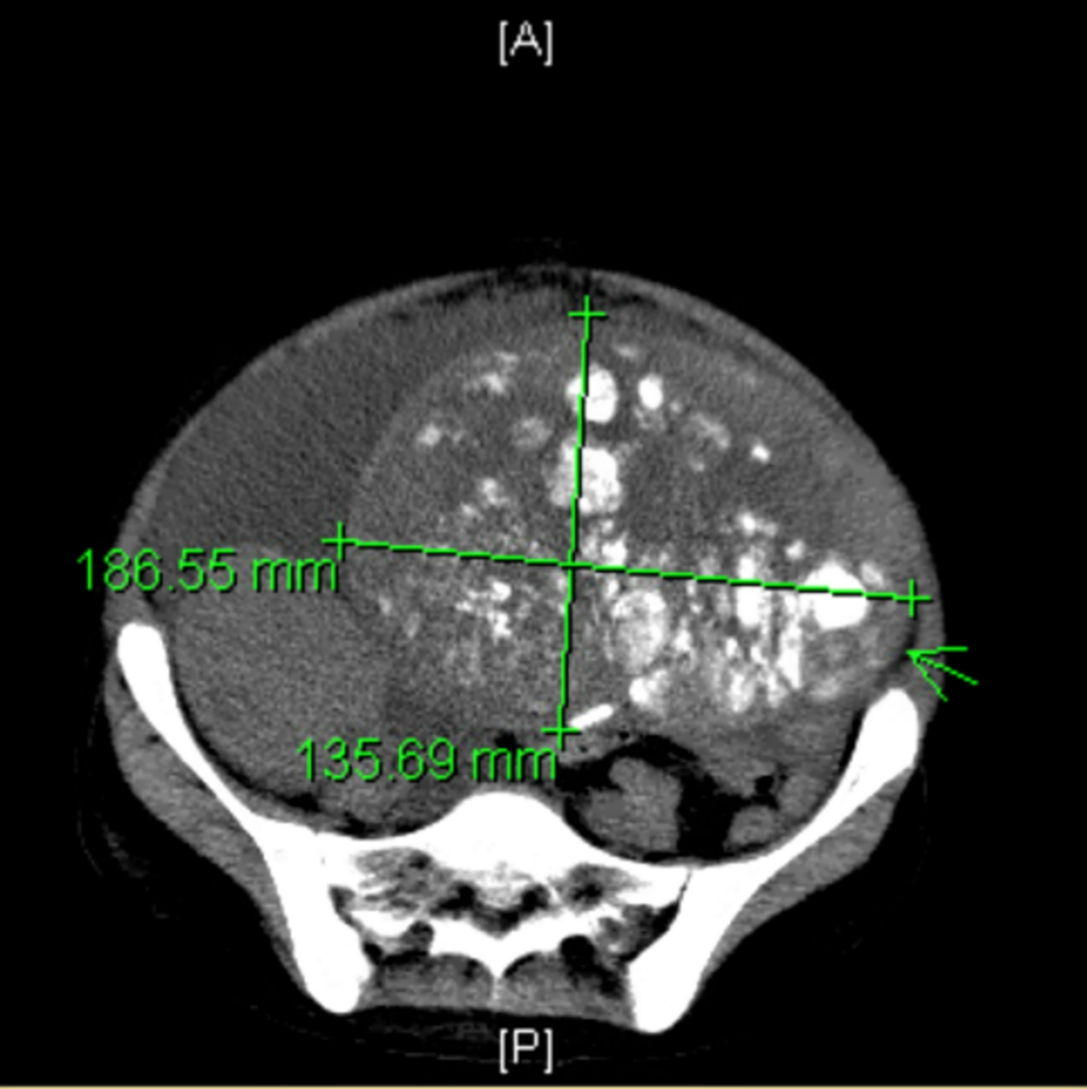
Abdominal mass showing multiple calcifications

**Figure 2:**
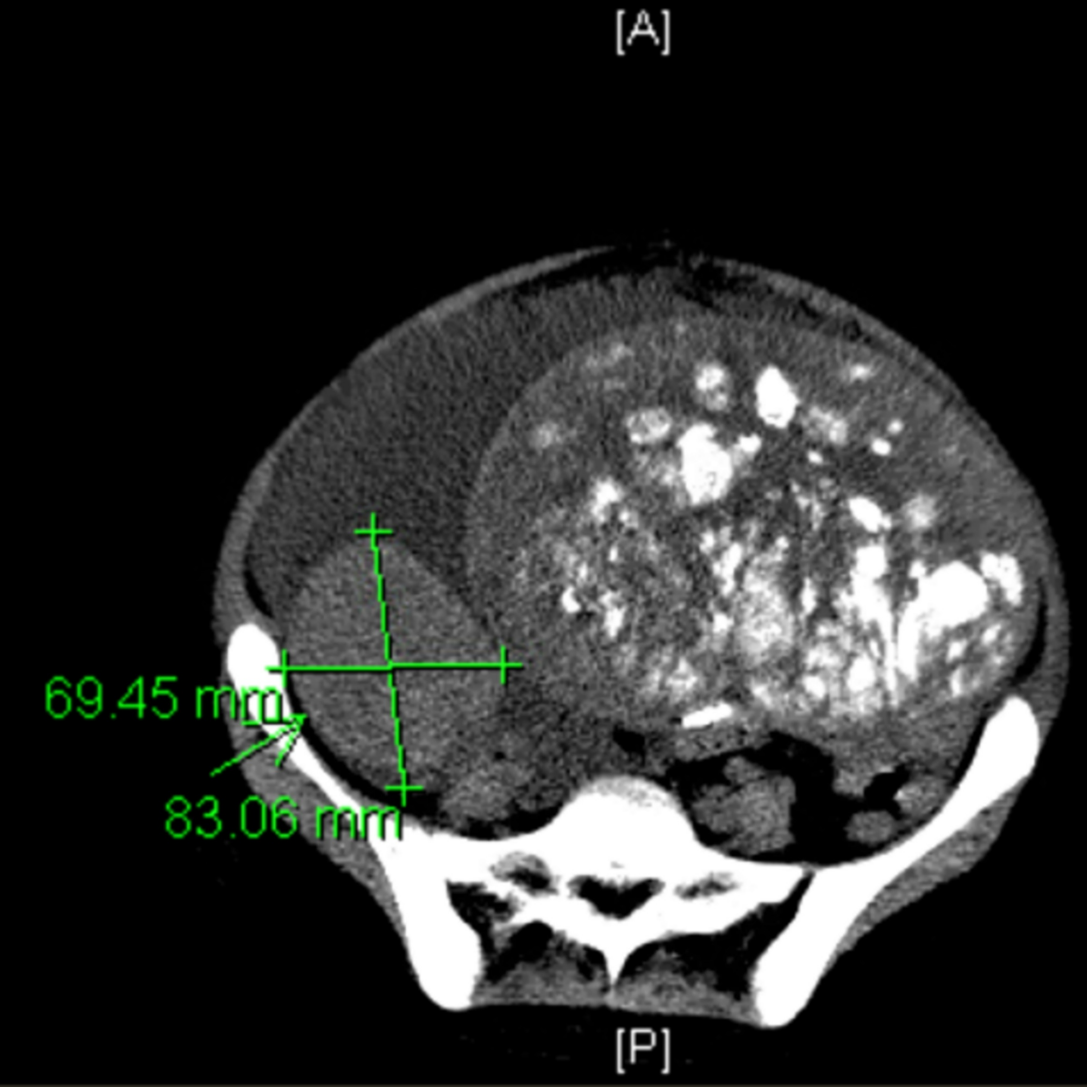
CT scan of the abdomen showing a right-sided pelvic mass

Pelvic ultrasound unveiled bilateral ovarian masses, so an MRI abdomen and a CT chest abdomen pelvis were obtained. These imaging studies showed bilateral ovarian masses, right-sided pleural effusion, large bilateral renal stones, hydronephrosis, a markedly enlarged 13.6x 18.7x 20.7 cm left pelvic mass, containing innumerable rounded calcifications (Figure 1), and a solid right pelvic mass measuring 8.3 x 7x 9.2 cm (Figure 2).

**Figure 3:**
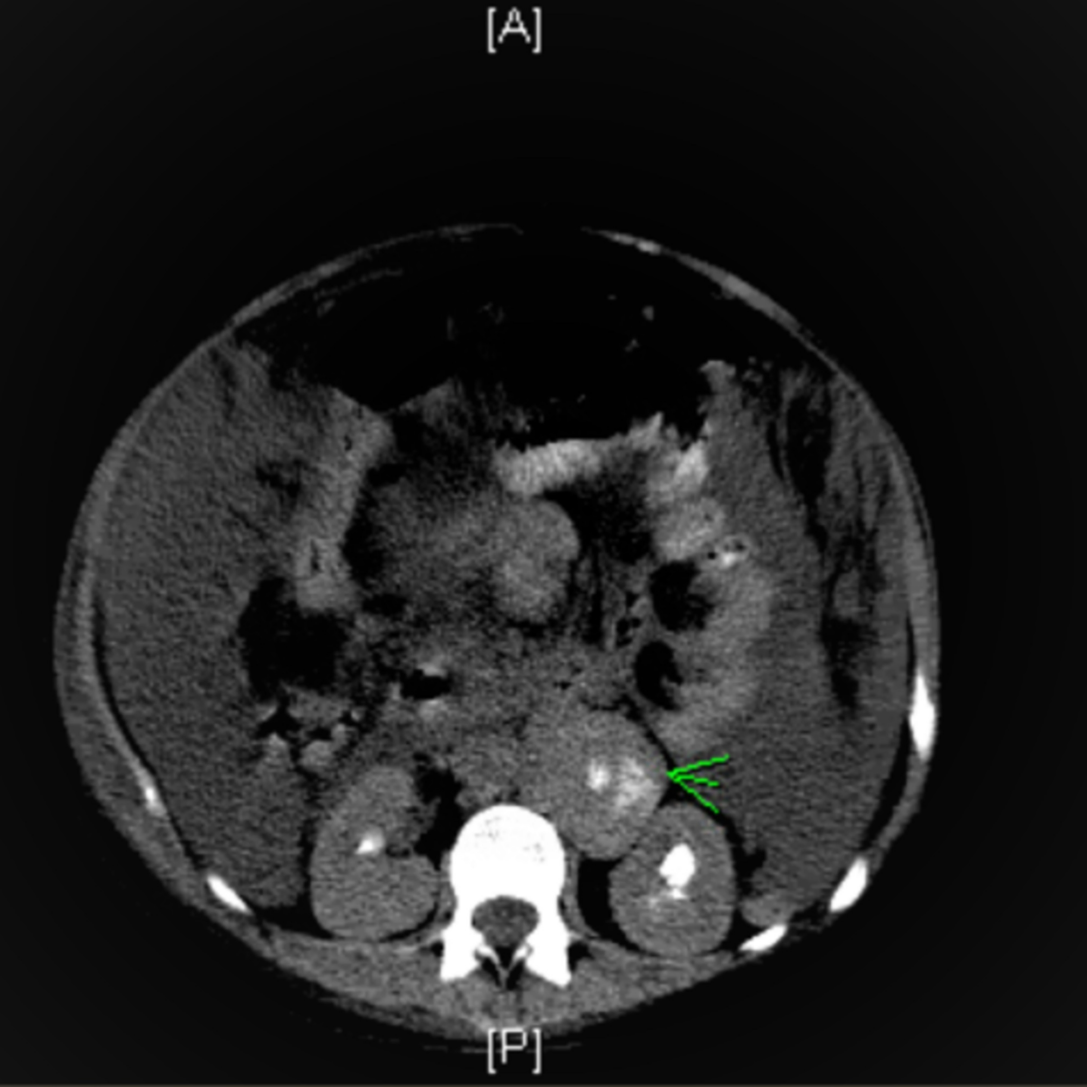
CT scan of the abdomen with the enlarged retroperitoneal lymph nodes containing calcifications

It also revealed ascites, diffuse omental infiltration, and retroperitoneal and periaortic lymphadenopathy containing the same calcific foci measuring up to 5.2 x 5cm (Figure 3).

With severe underlying hypercalcemia, a concern was raised for a squamous cell carcinoma arising from a mature cystic teratoma or a small cell carcinoma of the ovary. Paracentesis and thoracentesis were performed, draining 2.5 and 2.2 liters of fluid, respectively. Since the right pleural effusion was causing the mediastinal shift to the left side, a right-sided chest tube was also placed. Cytology results from thoracentesis and paracentesis were negative for malignant cells.

For hypercalcemia, aggressive intravenous (IV) hydration was provided with normal saline, which was then changed to half normal saline along with bicarbonate due to the metabolic acidosis. She also received 90 mg of pamidronate intravenously over two hours and was then continued on subcutaneous calcitonin. It was postulated that large renal stones might have been caused by hypercalcemia. Furosemide 20 mg IV was initially given to promote calciuria but was then stopped based on the fact that the theoretical calciuric effect is outweighed by the diuretic effect that perpetuates hypercalcemia by causing further dehydration.

**Figure 4:**
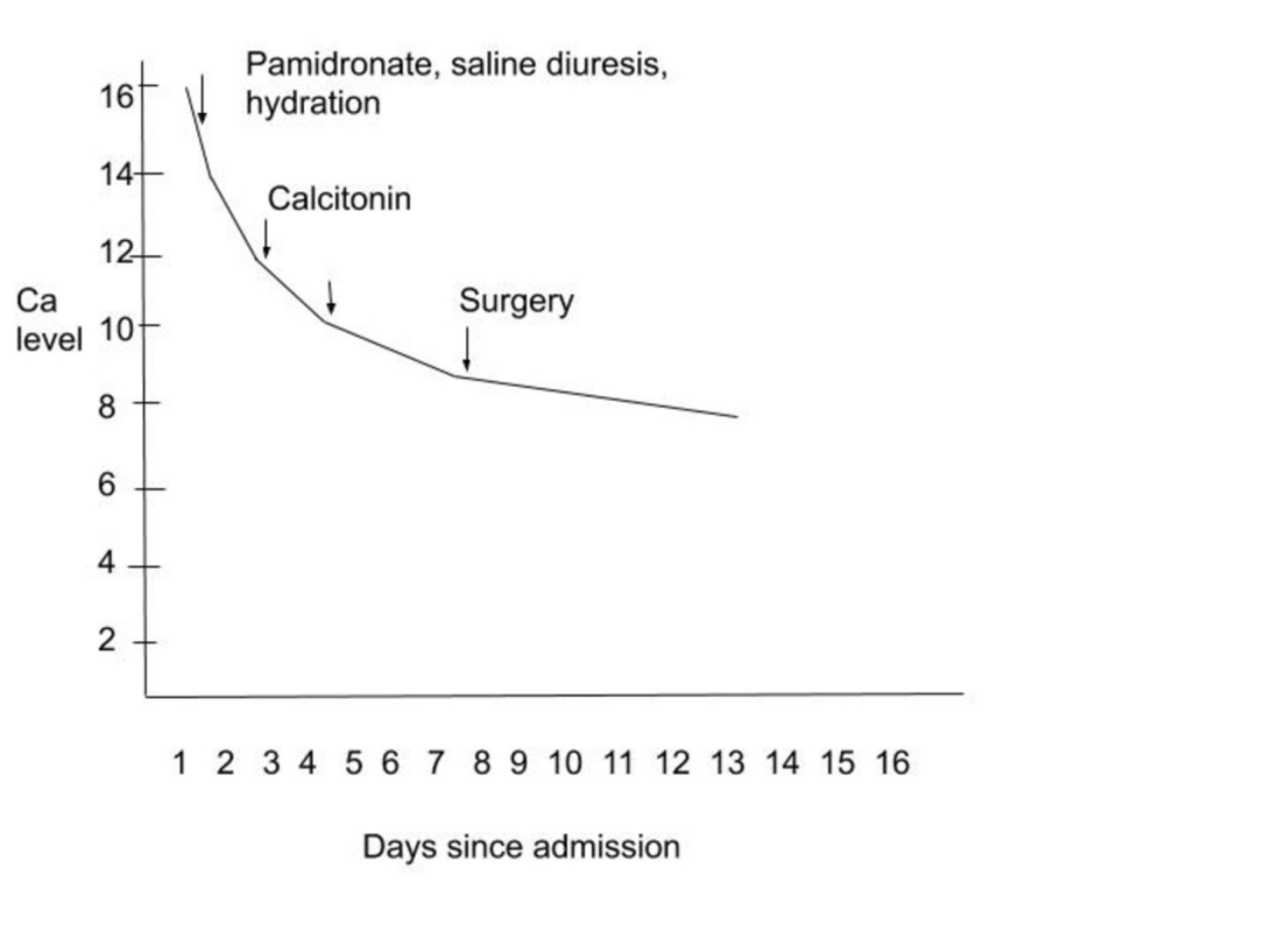
Down-trending levels of calcium after treatment

The graphical representation of the down-trending calcium levels with treatment is shown in Figure 4.

At Day 3 of her admission, it was considered to add prednisone for treating hypercalcemia to decrease intestinal calcium absorption. Total parenteral nutrition (TPN) was started after the first few days due to her severe malnutrition and inability to eat. Meanwhile, she also developed coagulopathy as a result of malnutrition and vitamin K deficiency and the international normalized ratio (INR) started to rise >10, and she got 10 mg of IV vitamin K. As the vitamin D levels were in the high normal range, despite low parathyroid hormone (PTH) levels, it was postulated that hypercalcemia was secondary to the ectopic production of 1 alpha-hydroxylase. Usually, hypercalcemia should cause a negative feedback effect on vitamin D production, and its levels should be low. A high normal value suggests vitamin D activation in an abnormal manner, which could happen because of the 1 alpha-hydroxylase production from the tumor. This mechanism mainly leads to the enteral absorption of calcium. High-dose corticosteroids can be helpful to decrease enteral absorption, but since our patient had very poor oral intake and was on TPN, this intervention was not very helpful. Calcitonin treatment was continued.

**Figure 5:**
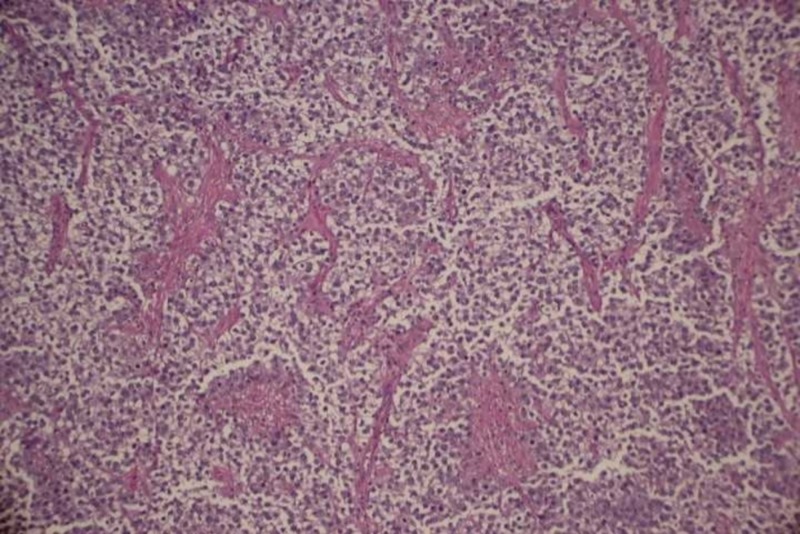
Low power field view of dysgerminoma

**Figure 6:**
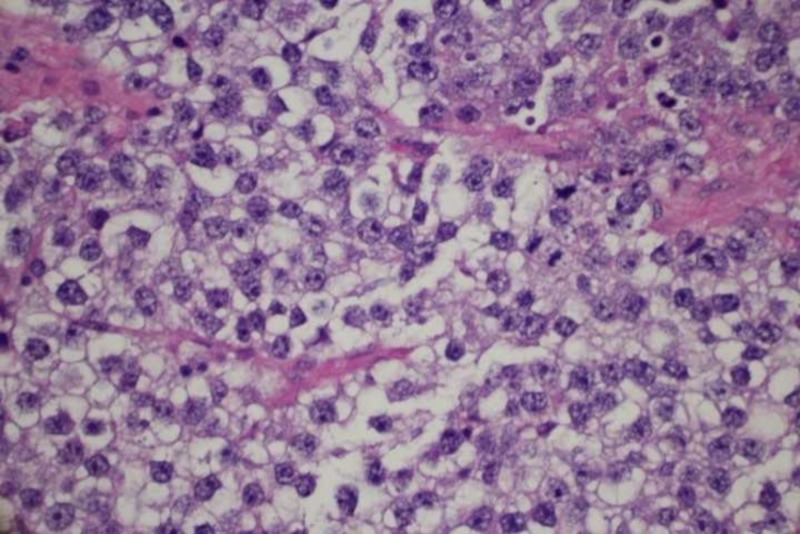
High power field view of dysgerminoma

On Day 8 of her admission, she underwent exploratory laparotomy, and we removed a 25-cm mass of the left ovary, invading the fallopian tube and pelvic lymph nodes, and an 11-cm mass of the right ovary. Procedures performed included exploratory laparotomy, bilateral salpingo-oophorectomy, pelvic and extensive paraaortic lymph node dissection, omental biopsy, and takedown of the falciform ligament. The pathology results revealed bilateral ovarian dysgerminomas, with granulomatous inflammation and lymphatic involvement. Pathology images of the dysgerminomas showed a uniform population of round cells, separated by fibrous strands and infiltrated by T lymphocytes, shown in Figure 5 in the low power field and Figure 6 in the high power field.

**Figure 7:**
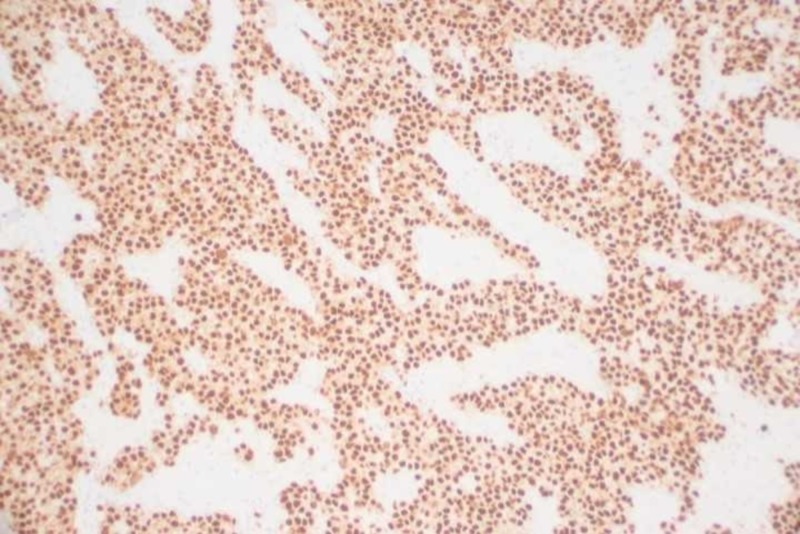
Optical coherence tomography 3/4 stain (OCT 3-4) positive immunohistochemical staining of dysgerminoma

The Immunohistochemical staining of her dysgerminoma was optical coherence tomography 3/4 stain (OCT3/4) positive, as shown in Figure 7.

The calcium level started to decrease dramatically after tumor resection. Calcitonin was stopped and the patient gradually weaned off TPN after the resumption of enteral nutrition in two weeks. The chest tube was removed 13 days after the surgery. The patient had an uneventful postoperative course and was eventually discharged home in a stable condition. She has not developed complications and recurrence of the disease.

## Discussion

While malignancy-associated hypercalcemia is a common finding among 20% to 30% of adult patients with breast and lung cancer and multiple myeloma, it happens in less than 5% of female genital tract malignancies [1]. The three most common mechanisms associated with hypercalcemia are: (a) local osteolytic hypercalcemia, most commonly observed in breast cancer, multiple myeloma, and lymphoma; (b) humoral hypercalcemia of malignancy (HHM) caused by systemic secretion of parathyroid hormone-related peptide (PTHrP), most commonly caused by squamous, renal, and ovarian cancer. Other humoral factors responsible for hypercalcemia in several malignant tumors include prostaglandin, tumor necrosis factor, osteoclast activating factor, transforming growth factor. Among them, PTHrP and 1,25-dihydroxy vitamin D have been reported as the humoral factors of ovarian dysgerminoma [2]. In almost all cases (95%) of female genital tract malignancies, hypercalcemia is caused by humoral hypercalcemia of malignancy (HHM). Ovarian cancer is the most common female genital tract malignancy that is associated with HHM. HHM related to the female genital tract is most often (80%) caused by PTHrP. Dysgerminoma is the second most common ovarian neoplasm to be associated with HHM [3].

Radhakrishnan et al. have mentioned a report from a gynecological oncology service, where seven of 34 consecutive women with gynecological cancer had hypercalcemia. This report suggested that hypercalcemia was more common with squamous cancer and in women with a large tumor burden, often as a result of osteolytic metastases. In the absence of liver and bone metastases, a hormonal cause was postulated. Biochemical abnormalities were those of hyperparathyroidism, but PTH levels were low, as in our patient [4-5].

From our extensive literature search, we found that there have been only six previously reported cases of dysgerminoma, which were associated with hypercalcemia [2,6-10]. The list of these cases is given below in Table 2. Among these cases, the association is mainly shown between hypercalcemia and dysgerminoma, but the contribution of 1,25 dihydroxyvitamin D to this problem is yet to be fully defined. We found only one study that analyzed the role of 1,25 dihydroxyvitamin D in contribution to hypercalcemia [11].

**Table 2:** Characteristics of previously reported cases of dysgerminoma-related hypercalcemia

S. No	Author	Age/sex	Presentation	Diagnosis	Treatment	complications	outcome
1.	Okoye Bo et al [6]	14/F	Abdominal distension	Dysgerminoma, hypercalcemia	Excision of tumor	N/A	recovery
2.	Sandra M. Allbery et al [2]	13/F	Anorexia and abdominal enlargement	Ovarian dysgerminoma, high PTH related hypercalcemia	Pamidronate, calcitonin, tumor excision	Renal medullary calcifications due to hypercalcemia	recovery
3.	Nelken Robert P. et al [7]	10.5/F	Calcified abdominal mass, weight loss, polyuria, polydipsia	Calcified intra-abdominal dysgerminoma	Tumor excision	Hypercalcemia	recovery
4.	Fleischhacker DS et al [8]	19/F	Polyuria, polydipsia, cachexia	Right ovarian dysgerminoma	Surgical excision	N/A	recovery
5.	Anstey A et al [9]	17/ F	Nausea, vomiting, constipation	Bilateral dysgerminomas, normal PTH (like our case)	Surgical excision	Malnutrition due to nausea and vomiting	recovery
6.	Stewart AF et al [10]	15/ F	Weight loss	Dysgerminoma of L ovary	Surgical resection	Para Aortic lymph node involvement, no bone metastases	recovery

1,25 dihydroxyvitamin D can cause hypercalcemia by a combination of enhanced osteoclastic bone resorption and enhanced intestinal absorption of calcium [12]. In one investigation, it was shown that the inflammatory mechanism associated with dysgerminomas is the underlying cause of the increased activity of 1 alpha-hydroxylase, catalyzing the synthesis of the active form of 1,25 dihydroxyvitamin D3 [12].

Ovarian dysgerminomas grow rapidly, and patients often present with abdominal enlargement, which was the presentation in our case as well. They can produce various markers, e.g., placental-like alkaline phosphatase, LDH, and beta-hCG, which can be used to monitor disease activity [13]. In our patient, LDH and CA 125 levels were quite high, although beta HCG, CEA, and CA 19-9 levels were normal.

To investigate the etiology of hypercalcemia associated with dysgerminoma without bone metastasis, we suggest measuring the levels of PTH, PTHrP, 25-hydroxyvitamin D, and 1,25-dihydroxyvitamin D. If possible, the analysis of 1α-hydroxylase activity in tissue samples may be useful. High or upper normal levels of 1,25-dihydroxyvitamin D in the absence of high PTH levels suggest vitamin D production independent of the effect of PTH. Normally, when PTH is high, 1,25 dihydroxyvitamin D levels become high, while low PTH leads to a decrease in its levels. Thus, in the setting of low PTH, a normal or high normal 1,25 dihydroxyvitamin D level is considered to be inappropriately normal and suggests ectopic vitamin D production. This was exactly the case with our patient. 1 alpha-hydroxylase is the enzyme responsible for the production of the active form of vitamin D, i.e., 1,25 dihydroxyvitamin D. Activity of 1 alpha-hydroxylase was evaluated in a collection of 12 dysgerminomas. RT-PCR analyses indicated that mRNA for 1 alpha-hydroxylase was increased 222-fold in dysgerminomas as compared to non-tumor ovarian tissue. Parallel enzyme assays in tissue homogenates showed that dysgerminomas produced fivefold higher levels of 1,25 dihydroxyvitamin D compared to normal ovarian tissue. Based on immunolocalization studies, Evans et al. demonstrated that 1 alpha-hydroxylase was expressed by both the tumor cells and the macrophages within the inflammatory cell infiltrate associated with dysgerminomas [11]. Unfortunately, 1 alpha-hydroxylase assay was not performed in our patient although low PTH and PTHrP with high normal 1,25 dihydroxyvitamin D level suggested that hypercalcemia originated from ectopic 1,25 dihydroxyvitamin D production.

Rehydration is the first step in the treatment of hypercalcemia patients. Patients can be very dehydrated due to the nephrogenic diabetes insipidus induced by hypercalcemia and decreased oral hydration from anorexia and nausea. Loop diuretics may also be added to increase calcium excretion once adequate hydration has occurred. There are various drugs that are effective at lowering serum calcium levels through other means, such as calcitonin and pamidronate. However, HHM is extremely resistant to control by these medical treatments. Therefore, it is most important to treat the tumor itself [2]. There was a retrospective study on patients with a median age of 9.5 years, which showed a great prognosis after tumor resection. Relapse free survival rate was 93.4%, and overall survival was 98.3% [13]. Our patient responded very well to the aggressive IV hydration, pamidronate, furosemide, and subcutaneous calcitonin, but the definitive improvement in calcium appeared after surgery. The use of loop diuretics is tricky because they are helpful only if the patient is sufficiently hydrated; otherwise, their diuretic effect can perpetuate hypercalcemia by causing further dehydration. Another important challenge lies in the utility of corticosteroids. Their benefit in decreasing gastrointestinal absorption of calcium is limited if the patient has restricted oral intake due to nausea and vomiting. Although our patient did receive furosemide and steroids, they were discontinued when they were not thought to be very effective. Hence, a case-based treatment plan should be devised based on the clinical progress of the patient. Further studies can be done to look for the benefits of steroids in decreasing the activity of 1 alpha-hydroxylase in tumors, as was seen in the case of granulomas.

## Conclusions

Hypercalcemia can be a complication associated with dysgerminomas. In rare cases, dysgerminomas can produce excessive amounts of 1, alpha-hydroxylase and 1, 25 dihydroxyvitamin D that can contribute to hypercalcemia. In such cases, aggressive hydration is usually required and steroids can be particularly helpful in reducing the 1 alpha-hydroxylase activity and decreasing the absorption of calcium via intestines. Resection of the tumor is the definitive treatment of hypercalcemia.

## References

[1] Stewart A (2005). Hypercalcemia associated with cancer. N Engl J Med.

[2] Allbery SM, Swischuk LE, John SD (1998). Hypercalcemia associated with dysgerminoma: case report and imaging findings. Pediatr Radiol.

[3] Piura B (2008). Hypercalcemia in malignancies of the female genital tract [Article in Hebrew]. Harefuah.

[4] Radhakrishna S, Haq S, Lofts F, Young MP, Barton DP (2001). Ovarian dysgerminoma presenting with hypercalcemia. BJOG.

[5] Gallion HH, van Nagell JR Jr, Donaldson ES, Powell DE (1988). Ovarian dysgerminoma: report of seven cases and review of the literature. Am J Obstet Gynecol.

[6] Okoye BO, Harmston C, Buick RG (2001). Dysgerminoma associated with hypercalcemia: a case report. J Pediatr Surg.

[7] Nelken RP, Nieburg PI, Bergstrom WH, Richman RA (1979). Dysgerminoma presenting as a calcified abdominal mass with hypercalcemia. Pediatrics.

[8] Fleischhacker DS, Young RH (1994). Dysgerminoma of the ovary associated with hypercalcemia. Gynecol Oncol.

[9] Anstey A, Gowers L, Vass A, Robson AO (1990). Ovarian dysgerminoma presenting with hypercalcemia. Case report and review of the literature. Br J Obstet Gynaecol.

[10] Stewart AF, Romero R, Schwartz PE, Kohorn EI, Broadus AE (1982). Hypercalcemia associated with gynecologic malignancies: biochemical characterization. Cancer.

[11] Evans KN, Taylor H, Zehnder D (2004). Increased expression of 25-hydroxyvitamin D-1alpha-hydroxylase in dysgerminomas: a novel form of humoral hypercalcemia of malignancy. Am J Pathol.

[12] Hibi M, Hara F, Tomishige H (2008). 1,25-dihydroxyvitamin D-mediated hypercalcemia in ovarian dysgerminoma. Pediatr Hematol Oncol.

[13] Yang C, Wang S, Li CC, Zhang J, Kong XR, Ouyang J (2011). Ovarian germ cell tumors in children: a 20-year retrospective study in a single institution. Eur J Gynaecol Oncol.

